# Research Advances in Myocardial Infarction Repair and Cardiac Regenerative Medicine via the Notch Signaling Pathway

**DOI:** 10.31083/RCM26587

**Published:** 2025-03-19

**Authors:** Songyan Cai, Qingyuan Dai

**Affiliations:** ^1^Department of Cardiology, First Affiliated Hospital of Kunming Medical University, 650032 Kunming, Yunnan, China; ^2^Department of Physical Examination for Cadres, First Affiliated Hospital of Kunming Medical University, 650032 Kunming, Yunnan, China

**Keywords:** myocardial infarction, Notch signaling pathway, cardiac injury repair, cardiac regenerative

## Abstract

Acute myocardial infarction is myocardial necrosis caused by acute and persistent ischemia and hypoxia in the coronary artery and severely affects public health. Recently, stem cell research has presented transformational developments in treating myocardial infarction. The Notch signaling pathway plays a crucial role in the post-myocardial infarction repair process and cardiac regenerative medicine. Additionally, the Notch signaling pathway can be involved in regulating the inflammatory response, myocardial fibrosis, oxidative stress, cardiomyocyte apoptosis, and cardiomyocyte regeneration after myocardial infarction. Moreover, the Notch signaling pathway is applied in cardiac tissue engineering. This review mainly elaborates on the research on the Notch signaling pathway in repairing myocardial infarction and cardiac regenerative medicine, aiming to provide a reference for treating acute myocardial infarction.

## 1. Introduction

The incidence and mortality of cardiovascular diseases rank first worldwide. As of 2021, cardiovascular diseases have promoted approximately 19.9 million deaths, among which the number of patients with ischemic cardiomyopathy amounts to 9.2 million [1]. Due to the limited regenerative capacity of human cardiomyocytes, pathological remodeling occurs in infarcted myocardial tissue, resulting in poor cardiac contractility and the progression to heart failure, which seriously impairs the health of patients. The current main therapeutic approaches can merely control symptoms and decelerate the process of pathological remodeling in the heart; however, these approaches cannot salvage the irreversible damage to the heart and result in an unsatisfactory long-term prognosis. Following the recent in-depth research in cardiac regeneration, new perspectives have been offered to treat myocardial infarction. The Notch signaling pathway is widely and highly conserved in organisms and participates in cell development, tissue formation, and organ construction in various organisms ranging from Drosophila to humans [2]. Past studies have indicated that the Notch signaling pathway is intimately associated with myocardial infarction. On the one hand, the abnormal activation or inhibition of the Notch signaling pathway participates in post-myocardial infarction repair. On the other hand, the Notch signaling pathway can also facilitate the differentiation of stem cells into cardiomyocytes and enhance the regenerative capacity of cardiac tissue. The application prospects of this pathway in cardiac tissue engineering offer novel possibilities for cell replacement therapy in cardiac diseases. Therefore, in-depth exploration of the action mechanisms of the Notch signaling pathway in cardiac repair and regenerative medicine following myocardial infarction not only assists us in better comprehending the pathogenic mechanisms of cardiac diseases but also furnishes theoretical grounds and experimental bases for developing new therapeutic approaches. By summarizing and analyzing the recent research advancements regarding the Notch signaling pathway in repairing the heart following myocardial infarction and cardiac regenerative medicine, this review aims to provide beneficial references and inspiration for researchers in related fields.

## 2. Notch Signaling Pathway

The Notch signaling pathway is prevalently detectable in vertebrates and invertebrates, demonstrating remarkable evolutionary conservation. Moreover, the Notch signaling pathway exerts a pivotal regulatory role in differentiating and developing cells, tissues, and organs by mediating intercellular interactions among adjacent cells [3]. This pathway includes four Notch receptors (Notch1–4) and five ligands (Jagged1, Jagged2, Delta-like1, Delta-like3, and Delta-like4). When transmembrane Notch receptors are activated by Jagged and Delta ligand family members on the cell surface, they are cleaved by two protease enzymes, releasing the Notch intracellular domain (NICD). The NICD translocates into the nucleus, where its N-terminal RBP-Jκ (recombination signal binding protein for immunoglobulin kappa J region)-associated molecule (RAM) region and ankyrin repeat (ANK) domain bind transcription factor CSL (CBF1/Su(H)/Lag-1, also known as RBP-Jκ) and recruit coactivator Mastermind-like-1 (MAML1), thereby activating the transcription of downstream target genes. As research into the Notch signaling pathway has expanded, it was found that the Notch signaling pathway plays a crucial role in heart development and disease [4]. Numerous studies have also indicated that the Notch signaling pathway participates in the development of inflammatory responses and myocardial fibrosis after myocardial infarction [5, 6], as well as in regulating oxidative stress and cardiomyocyte apoptosis [7]. Furthermore, studies have demonstrated that the Notch signaling pathway induces the proliferation and differentiation of cardiomyocytes, promotes cardiomyocyte regeneration, and is utilized in cardiac tissue engineering [8, 9, 10].

## 3. Function of the Notch Signaling Pathway in the Repair of Myocardial Infarction 

### 3.1 Pathophysiological Processes Following Myocardial Infarction

A sharp decrease or interruption in coronary artery blood supply results in severe and persistent myocardial ischemia, thereby causing myocardial ischemic necrosis. In the early stage of myocardial infarction, cardiomyocytes undergo necrosis or apoptosis. The damaged cardiomyocytes will trigger an inflammatory response, and multiple types of inflammatory cells infiltrate the infarcted myocardial area and phagocytose necrotic cells and extracellular matrix debris [11]. During myocardial ischemia-reperfusion, oxidative stress can result in ischemia-reperfusion injury. The reactive oxygen species (ROS) generated within mitochondria are the major factors contributing to ischemia-reperfusion injury. ROS can alter the permeability of mitochondria, influence the function of the mitochondrial respiratory chain, induce dysfunction of cells and tissues, and, consequently, lead to cell death and myocardial fibrosis [12]. During the proliferation and repair phase, cardiac fibroblasts proliferate and secrete extracellular matrix proteins, forming fibrotic scars that replace dead myocardial cells. These tightly cross-linked fibrotic scars have a significant tensile strength that prevents rupture; however, as the left ventricular wall stress increases, left ventricular remodeling gradually occurs, leading to hypertrophy of cardiomyocytes in the infarction border zone, ventricular wall thinning, and ventricle dilation.

### 3.2 Role of Notch Signaling Pathway in Inflammation

The activation and resolution of inflammatory responses are related to the repair and ventricular remodeling processes following myocardial infarction. Either deficiency or excess of these responses can impact cardiac remodeling, thereby resulting in the deterioration of cardiac function in patients and influencing prognosis. The Notch signaling pathway is crucial to the inflammatory response after myocardial infarction [13, 14]. Neutrophils are the earliest immune cells to congregate in the heart after myocardial infarction. Moreover, neutrophils can phagocytose and eliminate necrotic cells and extracellular matrix debris in the infarcted zone and concurrently secrete proinflammatory cytokines to enhance the aggregation of other immune cells, such as monocytes [15]. Excessive neutrophil infiltration exacerbates tissue injury due to the excessive accumulation of inflammatory mediators and proteinases [16]. Thus, blocking the Notch signaling pathway can suppress the expression of vascular cell adhesion molecule 1 (VCAM1), thereby diminishing the infiltration of neutrophils. Furthermore, endomucin (EMCN) is a negative regulator of leukocyte adhesion. Inhibiting the Notch signaling pathway can curb neutrophil transendothelial migration by upregulating EMCN, thus modulating the inflammatory response [17, 18]. Meanwhile, the Notch signaling pathway also plays a role in activating macrophages [19]. The Notch1/2 signaling pathway activated by Delta-like ligands 1/4 (DLL1/4) can promote the expansion of macrophages by activating interferon regulatory factor (IRF4) [20]. Macrophages assume a crucial role in myocardial infarction [21]. Therefore, macrophages can differentiate into two different phenotypes depending on their activation state: proinflammatory M1 macrophages and reparative M2 macrophages. M1-type macrophages reach their peak at approximately day 3 [22], generating a significant amount of proinflammatory cytokines (interleukin (IL)-1, IL-6, tumor necrosis factor-α (TNF-α), proteases, etc.) and eliminating damaged cardiomyocytes and other cell debris [23]. The Notch signaling pathway promotes macrophages to polarize towards the proinflammatory M1 phenotype and enhances inflammatory responses. Notch- RBPJ selectively enhances the expression of the classical M1 macrophage gene subpopulation induced by Toll-like receptor 4 (TLR4), such as *IL12a*, *IL12b*, and *Nos2* [24]. Although M1 macrophages play a certain role in the inflammatory response after a myocardial infarction, excessive inflammation may exacerbate cardiac injury. As the disease progresses and inflammation persists, M1 macrophages polarize into M2 macrophages with a pro-repair function. M2 macrophages, as anti-inflammatory macrophages, release various anti-inflammatory factors to suppress excessive inflammation after myocardial infarction. Concurrently, M2 macrophages secrete multiple growth factors and extracellular matrix proteins to promote the formation of new blood vessels and repair and remodel damaged myocardial tissue [25]. A study has demonstrated that the Notch signaling pathway activated by DLL4 inhibits IL-4-mediated M2 macrophage differentiation and selectively promotes the early apoptosis of M2 macrophages [26]. Additionally, blocking the Notch signaling pathway can facilitate the M2 polarization of macrophages and enhance cardiac function by correcting the imbalance of fibrosis remodeling following myocardial infarction [27].

### 3.3 Role of the Notch Signaling Pathway in Cardiac Fibrosis

Cardiac fibrosis is a crucial pathological foundation for pathological cardiac remodeling in various cardiovascular diseases since M2 macrophages can secrete multiple profibrotic cytokines, such as transforming growth factor-β (TGF-β). When TGF-β binds to the receptor (TGFβRII, TGFβRI) on the surface of cardiac fibroblasts, the receptor phosphorylates the mothers against decapentaplegic homolog 2 (Smad2) and Smad3 proteins. The phosphorylated Smad2/3 proteins form a complex with Smad4, which is then translocated into the nucleus to regulate the expression of genes related to extracellular matrix components such as collagen (mainly type I and type III collagen), thereby facilitating the synthesis and secretion of collagen by fibroblasts [28]. In the early phase of acute myocardial infarction, cardiac fibrosis can sustain the structure and function of the heart and prevent cardiac rupture. Nevertheless, as collagen accumulates excessively, it influences the compliance of the cardiac structure, eventually resulting in ventricular remodeling and damage to the cardiac tissue function [29]. During the proliferation and repair stages following myocardial infarction, cardiac fibroblasts undergo phenotypic variations and functional modifications. The transformation of activated fibroblasts into myofibroblasts expressing α-smooth muscle actin (α-SMA) and secreting abundant cytokines and extracellular matrix is a key pathological process in myocardial fibrosis [30]. Existing studies have unveiled the potential interaction between Notch signaling and the TGF-β1/Smad3 signaling pathways. TGF-β1 levels increase significantly during myocardial infarction and promote myocardial fibrosis through the TGF-β1/Smad3 signaling pathway. In contrast, the Notch signaling pathway can suppress the TGF-β1/Smad3 signaling pathway to inhibit myocardial fibrosis and improve cardiac function during myocardial infarction, exerting a cardioprotective effect. It has been discovered that during the TGF-β1-induced transformation of cardiac fibroblasts into myofibroblasts (CMT), the expressions of Notch1, Notch3, and Notch4 decrease, and the downward trend corresponds to the increasing trend in α-SMA expression and collagen synthesis. Therefore, inhibiting Notch signal transduction might facilitate CMT [31]. Other studies have supported this viewpoint. It has been demonstrated that the the Notch signal inhibits the transformation of CMT by antagonizing the TGF-β1/Smad3 signal transduction, suppressing myocardial fibrosis after myocardial infarction [6, 32, 33]. The proprotein convertase subtilisin/kexin type 9 (PCSK9) inhibitor SBC-115076 inhibits cardiac fibroblasts from differentiating into myofibroblasts through the Notch1/hairy and enhancer of split 1 (Hes1) signaling pathway, thereby improving cardiac fibrosis and ventricular dysfunction after myocardial infarction [34]. However, some researchers have put forward distinct viewpoints and subsequent research has indicated that the Notch signaling pathway can initiate fibrosis by activating the transcription of α-SMA and the transformation of myofibroblasts, and when the Notch signaling pathway is inhibited, fibrosis can be prevented or existing fibrosis can undergo complete regression [35, 36]. Additionally, inhibition of the Notch signaling pathway can limit ventricular remodeling and improve cardiac function after myocardial infarction [9, 37]. The role of the Notch signaling pathway in cardiac fibrosis represents a complex, multifaceted, and finely regulated process. Moreover, the expression levels of different members in the Notch signaling pathway may be closely related to its role in cardiac fibrosis, the time points of action, and the interactive regulatory network with other signaling pathways. Further in-depth studies on the role of the Notch signaling pathway in myocardial fibrosis could have great potential for application.

### 3.4 Role of the Notch Signaling Pathway in Oxidative Stress and Cardiomyocyte Apoptosis

During myocardial ischemia-reperfusion, an excessive accumulation of ROS occurs in the mitochondria, thereby triggering oxidative stress. Oxidative stress induces lipid peroxidation in the cell membrane, DNA chain rupture and modifies the structure and function of proteins, and results in cellular dysfunction and death [38]. ROS can directly act on myocardial fibroblasts, activate signaling pathways such as mitogen-activated protein kinase (MAPK), induce fibroblast proliferation, and promote myocardial fibrosis [39]. Sirtuins constitute a class of nicotinamide adenine dinucleotid (NAD^+^)-dependent deacetylases that significantly regulate mitochondrial function and antioxidant capacity. Under oxidative stress conditions, the depletion of NAD^+^ might reduce the activity of the sirtuins, influencing mitochondrial function and ROS scavenging and exacerbating myocardial fibrosis [40]. Furthermore, oxidative stress is capable of inducing inflammatory responses and cell apoptosis. Conversely, the release of inflammatory factors has been shown to increase stimulation of the signaling pathways and aggravate oxidative stress-related damage [41, 42]. The activation of the Notch signaling pathway can enhance cell viability, suppress cardiomyocyte apoptosis, and decrease ROS accumulation, thereby alleviating the damage induced by reperfusion [43]. Studies have demonstrated that lncRNA *NONHSAT098487.2* expression is elevated after myocardial infarction. In H_2_O_2_-treated AC16 cardiomyocytes, overexpression of *NONHSAT098487.2* can alleviate H_2_O_2_-induced oxidative stress injury by activating the Notch signaling pathway. However, Notch signal transduction inhibition attenuates the protective effect of *NONHSAT098487.2* on cardiomyocytes [44]. Aldolase A (ALDOA) can participate in the cardiac stress response. ALDOA overexpression positively regulates the vascular endothelial growth factor (VEGF) to activate the Notch1 signaling pathway, thereby inhibiting oxidative stress and apoptosis induced by hypoxia/reperfusion (H/R) in H9C2 cells. Conversely, the Notch inhibitor, carvacrol, reverses the inhibition of cellular oxidative stress and apoptosis induced by ALDOA in H/R-induced cells [45]. You* et al*. [46] demonstrated that administering the delta-like non-canonical Notch ligand 1 (DLK1), a member of the epidermal growth factor-like family, to mice intraperitoneally promoted neovascularization through the Notch1 signaling pathway, thereby ameliorating heart failure. Mechanistic studies also indicated that recombinant DLK1 (rDLK1) facilitated anti-apoptosis, proliferation, migration, and tube formation of endothelial progenitor cells (EPCs) via the Notch1 signaling pathway in EPCs cultured under hypoxia and serum-free conditions. Furthermore, rDLK1 significantly reduced intracellular and mitochondrial ROS, increased adenosine triphosphate (ATP) content and mitochondrial membrane potential, downregulated the expression of the short isoform of optic atrophy 1 (OPA-1) and upregulated the expression of mitochondrial fusion proteins (MFN1/MFN2/S-OPA1). However, a study suggests inhibiting the Notch pathway may alleviate the damage to cardiomyocytes induced by H/R [47]. Additionally, curcumin possesses anti-inflammatory, antioxidant, and cardioprotective functions [48]; curcumin can decrease the ROS level and the apoptosis rate of cardiomyocytes induced by H/R in H9C2 cells by suppressing the Notch signaling pathway [47].

## 4. The Application of the Notch Signaling Pathway in Cardiac Regenerative Medicine 

### 4.1 Notch Signaling Pathway Function in Cardiomyocyte Regeneration 

Recently, in-depth research on stem cell therapy focusing on cardiac regeneration has offered a novel idea for treating myocardial infarction. The Notch signaling pathway assumes a significant role in the study of cardiac regenerative medicine. The activation of the Notch signaling pathway is of paramount importance for regulating the proliferation of early postnatal cardiomyocytes. Nemir *et al*. [49] cultivated transgenic mice that overexpressed the Notch ligand Jagged1 on the surface of cardiomyocytes to activate Notch signaling in adjacent cardiomyocytes and non-cardiomyocytes. The activated Notch signaling pathway can sustain the proliferation of postnatal cardiac progenitor cells and cardiomyocytes and increase the number of cardiomyocytes in adult mice. Comparatively, non-cardiomyocytes isolated from pressure-induced hypertrophic hearts demonstrate the activation of the Notch signaling pathway. Meanwhile, *in vitro* experiments indicate that blocking the Notch signaling pathway can stimulate non-cardiomyocyte transdifferentiation into cardiomyocytes in pressure-induced hypertrophic hearts [9]. Hypoxic stress can stimulate the expression of Jagged1 in cardiomyocytes and promote the early cardiomyocyte differentiation of cardiomyocyte stem cells (CSCs) co-cultured with cardiomyocytes via the HIF-1α/Jagged1/Notch signaling pathway [50]. When mesenchymal stem cells (MSCs) were co-cultured with the immature cardiomyocytes of newborn mice, the MSCs regulated the proliferation of cardiomyocytes via the Notch-1/Jagged-1 pathway [51].

Moreover, Zebrafish can fully regenerate after myocardial injury. Meanwhile, studies have indicated that Notch signaling is activated in the atrial endocardium following ventricular ablation, allowing differentiated atrial cardiomyocytes to transdifferentiate into ventricular cardiomyocytes, thereby contributing to the ventricular regeneration of the zebrafish heart [52]. Cardiac mesenchymal stem cells (C-MSCs) are a novel subpopulation of MSCs derived from cardiac tissue; the Notch1 signaling pathway is important for C-MSCs to promote cardiac regeneration. EVs secreted by Notch1-overexpressing C-MSCs can effectively prevent cell death after myocardial infarction, promote angiogenesis and CM proliferation, and restore cardiac function [8]. Other studies have shown that the Notch inhibitor DAPT can enhance the transformation of mouse fibroblasts into induced cardiomyocytes through transcription factors GATA-binding factor 4 (GATA4), Heart-and neural crest derivatives-expressed protein 2 (HAND2), myocyte enhancer factor 2C (MEF2C), T-box transcription factor 5 (TBX5) [53].

In recent years, multipotent stem cell-derived cardiomyocytes have drawn increasing attention and experienced extensive applications in cardiovascular development, disease modeling, and regenerative medicine. During the differentiation process of multipotent stem cell-derived cardiomyocytes, the activation of the Notch signaling pathway exerts both positive and negative regulatory influences on cardiomyogenesis. During the differentiation of human embryonic stem cells (hESCs) into cardiomyocytes, members of the miR148A family can facilitate the differentiation of hESCs into lateral mesoderm and cardiomyocyte precursor cells by targeting the DLL1–Notch signaling pathway. The deficiency in the miR148A family (miR148A-TKO) promotes a decrease in the proportion of cardiomyocyte differentiation. Meanwhile, the additional knockout of DLL1 can reverse the inhibitory effect of miR148A-TKO hESCs on the differentiation of cardiomyocytes [54]. Ye* et al*. [55] utilized CRISPR/Cas9 genome editing technology to create multiple Notch1 knockout (N1KO) human induced pluripotent stem cell (iPSC) lines. Notch1 deficiency significantly downregulated the ventricular cardiomyocyte-specific genes encoding myosin regulatory light chain 2 ventricular muscle isoform 2 (*MYL2*) and Iroquois-class homeodomain protein IRX4. Conversely, Notch1 knockout enhanced the expression of atrial-specific genes such as nuclear receptor *NR2F2*, potassium channel *KCNJ3*, and atrial isoform *MYL7*. When iPSCs differentiate into cardiomyocytes, the simultaneous overexpression of nucleosome assembly protein 1-like protein 1(NAP1L1) can enhance γ-secretase activity, the expression of NICD, and downstream gene expressions, thereby inhibiting the differentiation process. However, administering the γ-secretase inhibitor, DAPT, can markedly inhibit the production of NICD and nullify the inhibitory effect of NAP1L1 overexpression on mesoderm induction and cardiomyocyte differentiation [56]. Overexpression of the secreted frizzled-related protein 4 (SFRP4), a member of the Wnt family, has been demonstrated to augment the generation of Notch1 and Hes1, thereby suppressing the cardiac differentiation of P19CL6 cells [57]. Furthermore, other studies have indicated that the Notch pathway exerts a biphasic effect in cardiomyogenesis, which is conducive to the differentiation of early cardiomyocytes but inhibits that of late cardiomyocytes. The addition of DAPT in the late stage intensifies the inhibition of endogenous Notch activity, thereby enhancing cardiomyogenesis [58]. In P19 cells, miR-375 can inhibit the proliferation and differentiation of cardiomyocytes by targeting Notch2, thus suggesting that Notch2 can facilitate the differentiation of cardiomyocytes [59].

### 4.2 Application Prospect of the Notch Signaling Pathway in Cardiac Tissue Engineering 

The Notch signaling pathway, functioning as a molecular mechanism that assumes a critical role in heart development and regeneration, has garnered significant attention regarding its application prospects in cardiac tissue engineering, which aims to substitute the damaged or dysfunctional tissues of human patients. Hydrogels offer a platform for the three-dimensional culturing of cardiovascular cells. Self-assembling peptides (SAPs) are a class of hydrogels composed of alternating hydrophilic and hydrophobic amino acids that self-assemble at physiologic pH and osmolarity to form a hydrogel. In the 2% SAP hydrogel, the delivery of the peptide mimetic of Notch1 ligand Jagged1 (RJ) to the infarcted rat heart could enhance cardiac function and contractility, concurrently reduce fibrosis, increase the endothelial vascular area, and ameliorate the expression of ki67 in myocardial infarction [60]. Gerbin *et al*. [61] developed a hydrogel containing the Notch ligand Delta-1 and utilized it as an injectable solution for transplanting human embryonic stem cell-derived cardiomyocytes (hESC-CMs) into the myocardium of infarcted rats. The activation of the Notch signaling pathway augmented the size and proliferation of hESC-CM grafts, thereby improving cardiac function after myocardial infarction. Wen* et al*. [62] manufactured a biodegradable poly-lactic-co-ε-caprolactone (PLCL) scaffold and applied it alongside a Notch agonist JAG peptide to enhance cardiomyocyte differentiation of human mesenchymal stem cells (hMSCs) and augment the regenerative potential of the scaffold as a bioengineered heart patch in myocardial infarction repair. DAPT was added to block the Notch signaling pathway in bioprinted cardiac tissue. Then, after 7 days of culture, the tissue development of bioprinted tissue treated with DAPT was enhanced, resulting in sudden peak fluorescence signals in calcium imaging and synchronous contraction. In addition, histological analysis revealed that DAPT-treated myocardial tissue exhibited increased α-actin expression, myocardial cell area, myocardial cell arrangement, and myocardial cell perimeter [10]. Tung* et al*. [63] developed a biomaterial capable of precisely manipulating the fate of stem cells and directing the Notch signaling. Subsequent relevant studies demonstrated that the activation of the Notch signaling pathway promotes ectodermal differentiation during the undifferentiated stage of pluripotent stem cells and augments cardiac differentiation during the KDR+ cardiovascular progenitor cell stage. Additionally, Notch activation can induce differentiated myocardial cell population proliferation.

## 5. Conclusions

Myocardial infarction is among the common cardiovascular disorders. The Notch signaling pathway can influence the development of inflammatory responses by altering the infiltration of neutrophils after myocardial infarction and modulating the phenotypes of macrophages. Myocardial fibrosis constitutes a crucial pathophysiological alteration during the repair process after myocardial infarction. Indeed, the regulation of fibrosis post-myocardial infarction holds significant importance for the prognosis of patients. The role played by the Notch signaling pathway in cardiac fibrosis renders it a potential therapeutic target for myocardial fibrosis. During the modulation of oxidative stress and cell apoptosis, the activation of Notch can inhibit cardiomyocyte apoptosis and mitigate oxidative stress, thereby enhancing cardiac function. Thus, by regulating the activity of the Notch signaling pathway, researchers can induce the differentiation of stem cells into cardiomyocytes. Following further in-depth exploration into the mechanism of action through which the Notch pathway functions in cardiac development and repair, it is anticipated that more innovative therapeutic strategies will be employed in cardiac tissue engineering. Nevertheless, disputes exist regarding the precise role of the Notch signaling pathway in post-myocardial infarction repair and cardiac regeneration. Additionally, the current evidence of the application of this pathway in clinical treatment is extremely limited; thus, further research is required to explore its latent value in the treatment of myocardial infarction.
